# Effects of 12‐week, non‐energy‐restricted dietary intervention with conventional yogurt οn appetite hormone responses of type 2 diabetic patients

**DOI:** 10.1002/fsn3.2606

**Published:** 2021-10-12

**Authors:** Amalia E. Yanni, Panagiotis Konstantopoulos, Kleio Kartsioti, Panagiota Binou, Vaios Τ. Karathanos, Artemis Chatzigeorgiou, Alexander Kokkinos

**Affiliations:** ^1^ Laboratory of Chemistry‐Biochemistry‐Physical Chemistry of Foods Department of Nutrition and Dietetics Harokopio University Athens Greece; ^2^ Laboratory of Experimental Surgery and Surgery Research School of Medicine National and Kapodistrian University of Athens Athens Greece; ^3^ Delta S.A. Athens Greece; ^4^ Diabetes Laboratory First Department of Propaedeutic Internal Medicine Laiko General Hospital School of Medicine National and Kapodistrian University of Athens Athens Greece

**Keywords:** ghrelin, glucagon‐like peptide‐1, peptide‐YY, type‐2 diabetes mellitus, yogurt

## Abstract

Hunger‐reducing effects and beneficial changes in gastrointestinal hormones have been reported, in overweight/obese individuals consuming dairy while yogurt takes pride of place due to its unique structure and composition. Although the contribution of yogurt to metabolic regulation has received growing attention, the research studies which examine its role on appetite are limited, especially regarding type 2 diabetes mellitus (T2DM) patients. The aim of the present study was to investigate the effects of non‐fat, conventional yogurt consumption on appetite hormone responses of T2DM patients following a non‐energy‐restricted diet. Overweight subjects participated in a 12‐week dietary intervention including 2 meals/day (2 × 200 g) of yogurt. At the beginning and the end of the intervention, a mixed meal tolerance test assessing the postprandial response of glucose, insulin, ghrelin, glucagon‐like peptide‐1 (GLP‐1), and peptide‐YY (PYY) was performed. Subjective appetite ratings were also evaluated. Area under the curve for glucose, insulin, ghrelin, GLP‐1, and PYY responses did not differ after the 12‐week intervention with yogurt (*p* > .05) as well as for subjective appetite ratings (*p* > .05). No significant differences were indicated at specific time points in any of the examined parameters. Regular consumption of non‐fat, conventional yogurt for 12‐week duration does not affect appetite hormone responses in overweight patients with T2DM following a non‐energy‐restricted diet.

## Graphical abstract

Twelve‐week conventional yogurt consumption does not affect appetite hormone responses of type II diabetic patients.

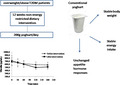

## 1 INTRODUCTION

Available data suggest that regular consumption of low‐fat dairy products has been associated with body weight management and reduced risk for type 2 diabetes mellitus (T2DM) especially for yogurt, although evidence from randomized clinical trials is lacking (Alvarez‐Bueno et al., 2019; Dougkas et al., 2011; Guo et al., 2019; Panahi & Tremblay, 2016; Yanni et al., 2020).

The amount of proteins and the composition of sugars in dairy products are considered major factors involved in appetite hormone regulation and satiety, energy intake, and glycemic control (Panahi et al., 2013). Hunger‐reducing effects, suppression of orexigenic hormone ghrelin, (Gilbert et al., 2011) and enhancement of anorectic peptides, glucagon‐like peptide‐1 (GLP‐1), and peptide‐YY (PYY) (Jones et al., 2013; Velhorst et al., 2008) in overweight/obese individuals consuming dairy under weight‐reducing programs have been reported.

Yogurt takes pride of place among dairy due to its unique structure and composition. Research data suggest that regular yogurt consumption promotes body weight management, a fact which can be attributed to the replacement of less healthy foods, the content of specific nutrients such as proteins and Ca, the presence of lactic acid bacteria as well as the structure of the food which can be used as a vehicle for ingredients such as dietary fibers and microorganisms, parameters which can contribute to the regulation of energy balance (Panahi & Tremblay, 2016; Tremblay et al., 2015).

However, the results of the clinical trials regarding the effects of yogurt on appetite regulation are scarce and remain inconsistent. For example, although it has been shown that plain yogurt with high protein content (i.e., 24 g/160 kcal) caused increased satiety and delay in request for the next meal, (Douglas et al., 2013) plain yogurt with high protein to carbohydrate ratio (PCR 2.30, 23.1 g proteins/141 kcal) did not cause any differences in subjective satiety ratings compared to strawberry yogurt with low PCR (0.79, 18.3 g protein/167 kcal) (El Khoury et al., 2014). Beyond the small number of studies, research design such as yogurt consumption under energy‐restricted diet or not; the study participants, high or low dairy consumers, men or women; the duration of the intervention, 12 weeks or higher; the type and the amount of yogurt, plain or fortified are parameters which significantly contribute to the variability which characterizes research results. As far as the implication of yogurt on appetite regulation of T2DM patients, there is severe lack of evidence. The investigation of the possible beneficial effects of regular yogurt consumption on gastrointestinal appetite‐regulating hormone responses after ingestion of a balanced meal is challenging, especially for overweight diabetic patients since body weight management is inseparably linked with disease management.

Under this point of view, the present study aims to investigate the effects of non‐fat, conventional yogurt consumption on appetite hormone responses of overweight T2DM patients who followed a non‐energy‐restricted diet. For this reason, eligible subjects participated in a 12‐week dietary intervention with yogurt, at the beginning and the end of which a mixed meal tolerance test was performed and gastrointestinal hormone responses and subjective appetite ratings were evaluated.

## 2 MATERIALS AND METHODS

### 2.1 Patients

The present study is a part of a larger trial on the effects of yogurt on diabetes outcomes in T2DM patients (Yanni et al., 2019). Subjects were recruited from the outpatient diabetes clinic of the 1st Department of Propaedeutic and Internal Medicine, Laiko General Hospital, Athens University Medical School. Inclusion criteria required overweight patients aged between 45 and 75 years with glycosylated hemoglobin (HBA_1c_) <8.5%, constant body weight, stable dietary habits, and oral medications (hypoglycemic, hypolipidemic, and antihypertensive) for the last 3 months before screening.

Exclusion criteria were lactose intolerance, allergies to milk or dairy products, history of cardiovascular, gastrointestinal, renal and endocrinological diseases, treatment for weight reduction, intake of supplements, and insulin therapy. Eligible subjects were informed in detail regarding the study protocol, and all the procedures before they gave voluntary informed written consent for participation.

Procedures involving human subjects were carried out in the Diabetes Laboratory of the 1st Department of Propaedeutic and Internal Medicine, Laiko General Hospital, Athens University Medical School. All protocols were reviewed and approved by the Institutional Review Board/Ethics Committee of Laiko General Hospital and the Bioethics Committee of Harokopio University of Athens. The study has been registered in clinicaltrials.gov as NCT03926806.

### 2.2 Study design

Eligible subjects (*n* = 12, 7 males/5 females) entered a 2‐week run‐in period in which they received dietary training by an expert dietitian and were instructed on a weight maintenance diet. All subjects were asked to keep their physical activity habits stable.

Patients were enrolled in a 12‐week dietary intervention in which they were asked to consume 2 servings per day (2 × 200 g) of non‐fat, conventional yogurt, once as mid‐morning snack and once as mid‐afternoon snack. Patients’ compliance was monitored by weekly telephone interviews. It was also checked by a 3‐day dietary food recall questionnaire and by a food frequency questionnaire which was filled at the beginning and the end of the 12‐week dietary intervention in order to evaluate dietary habits for the last 3 months (Yanni et al., 2019).

At the beginning and the end of the study, a mixed meal tolerance test was performed. Patients were asked to refrain from alcohol consumption and strenuous exercise 24 h before the study day while food intake was individually standardized. Subjects were invited to the laboratory between 07:00 and 09:00 am after 12 h of fasting. Before each session, physical examination and blood pressure measurement were performed. An intravenous catheter was placed in a forearm vein, and subjects were allowed to settle for 5–10 min before the first blood sample was drawn. They were then asked to consume a standardized meal consisting of 100 g rye bread, 56 g cheese (29% fat), 72 g turkey (2% fat), and 100 ml of orange juice (total energy content of the meal: 583 kcal; 52% carbohydrate, 29% protein, and 19% fat). Postprandial blood samples were collected at 30, 60, 90, 120, and 180 min after the first bite. Glucose, insulin, total ghrelin, GLP‐1, and PYY responses were evaluated.

Blood samples were collected while patients were in a sitting position according to the standard protocol. For the separation of plasma, vacutainers with K_3_EDTA as anticoagulant were used and the samples were centrifuged immediately (1000 *g* for 10 min at 4℃). Blood was collected in plain vacutainers, allowed to clot at room temperature for 30 min, and then centrifuged (3000 rpm for 10 min at 4℃) for the separation of serum. After isolation, plasma and serum samples were stored at −80℃ until analysis.

### 2.3 Blood analyses

Total ghrelin was determined as previously described (Yanni et al., 2017). In brief, the protease inhibitor phenylmethanesulfonyl fluoride was added in K_3_EDTA plasma immediately after isolation, and samples were then acidified with HCl and centrifuged for 10 min, at 3000 rpm and 4℃. A sandwich ELISA method (Human Ghrelin [Total] ELISA kit; Millipore) was used for the determination of total ghrelin at 0, 30, 60, 120, and 180 min. Total GLP‐1 and PYY were also detected in plasma and insulin in serum by sandwich ELISA methods using commercially available kits (Human Total Glucagon‐Like Peptide‐1 kit; Millipore, Human PYY [Total] ELISA kit; Millipore and Human Insulin ELISA kit; Millipore, respectively) at 0, 30, 60, 90, 120, and 180 min.

Glucose concentrations at 0, 15, 30, 45, 60, 90, 120, and 180 min were determined in plasma immediately by electrochemical method in an automated analyzer (ΥSI 2300 STAT PLUS).

At the beginning and the end of the dietary intervention, basal biochemical measurements were performed in serum by an automated biochemical analyzer (Yanni et al., 2019).

### 2.4 Subjective appetite ratings

Subjective appetite was assessed at the beginning and the end of the study during the mixed meal tolerance test. Ten cm visual analogue scales (VAS) anchored by terms “not at all” (0 cm) and “extremely” (10 cm) were used to evaluate hunger, fullness, and desire for the next meal. Specifically, VAS included three questions, that is, “How hungry do you feel,” “How full do you feel,” and “How great is your desire to eat.” Subjects were asked to complete the appetite questionnaire before the consumption of the meal (time 0) and at 30, 60, 90, 120, and 180 min postprandially.

### 2.5 Statistical analysis

Values are expressed as mean ± SEM. The Kolmogorov–Smirnov test was applied to examine normal distribution of variables. Areas under the curve (AUCs) for glycemic and insulinemic responses and for responses of total ghrelin, GLP‐1, and PYY were calculated applying the trapezoidal rule. AUC for VAS expressed as difference from preprandial values was calculated using iAUC ignoring the area above the *x* axis for “Hunger” and “Desire to eat” and below the *x* axis for “Fullness.” Paired samples Student's *t*‐test was used to compare the postprandial changes in blood variables and subjective appetite ratings and identify significant differences at specific time points. The same test was applied for AUC and iAUC values. Statistical significance was set at 0.05. For the analysis, SPSS 21.0 statistical software package was used.

## 3 RESULTS

### 3.1 Blood analyses

Subjects’ characteristics before intervention are presented in Table 1. Yogurt provided 110 kcal of energy, 19.2 g protein, 8.4 g carbohydrates, and 240 mg Ca per 200 g serving, and it was well‐tolerated by the patients. Compliance was checked when food items were provided to the participants and also by the 3‐day dietary food recall questionnaire and the food frequency questionnaire.

**TABLE 1:** Subjects’ characteristics before the intervention

Characteristic	
Age (y)	61.3 ± 1.2
Male/female	7/5
Body weight (kg)	82.5 ± 2.5
BMI (kg/m^2^)	29.0 ± 0.5
HbA_1c_ (%)	6.3 ± 0.2
Fasting plasma glucose (mg/dl)	137.9 ± 10.4

Note

Values are expressed as mean ± SEM.

Abbreviations: ΒΜΙ, body mass index; HbA_1c_, glycosylated hemoglobin.

**FIGURE 1:**
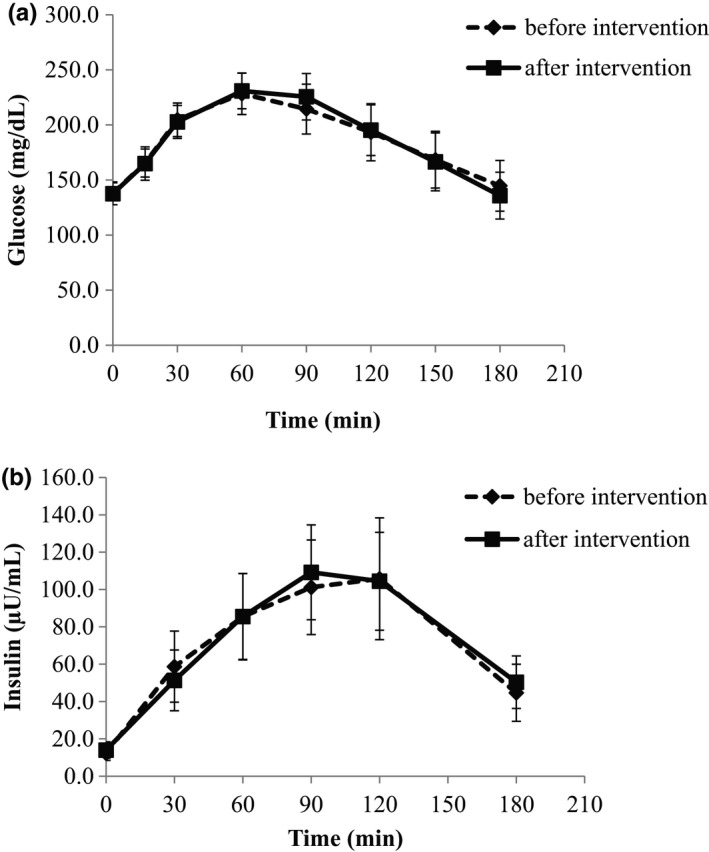
Postprandial glucose (a) and insulin (b) responses during the mixed meal tolerance test performed before and after the 12‐week dietary intervention with non‐fat, conventional yogurt. Values are presented as means ± SEM

Glucose and insulin responses to mixed meal tolerance test before and after the dietary intervention with non‐fat, conventional yogurt are demonstrated in Figure 1a,b, respectively. No significant differences were noticed before and after the intervention neither in specific time points nor in the AUCs (Table 2).

**TABLE 2:** AUC for glucose, insulin, ghrelin, GLP‐1, and PYY before and after the dietary intervention with non‐fat, conventional yogurt

AUC	Baseline	End
Glucose (mg/dl × 180 min)	10,152.47 ± 1987.45	10,375.17 ± 1720.15
Insulin (μU/ml × 180 min)	14,381.67 ± 4090.84	14,054.36 ± 3638.96
Ghrelin (pg/ml × 180 min)	110,677.00 ± 19,365.48	102,333.30 ± 15,259.73
GLP‐1 (pmol/L × 180 min)	8713.18 ± 927.88	8919.85 ± 984.70
PYY (pg/ml × 180 min)	21,057.24 ± 2192.48	20,504.51 ± 2941.56

Note

Values are expressed as mean ± SEM.

Abbreviations: AUC, area under the curve; GLP‐1, glucagon‐like peptide‐1; PYY, peptide‐YY.

**FIGURE 2:**
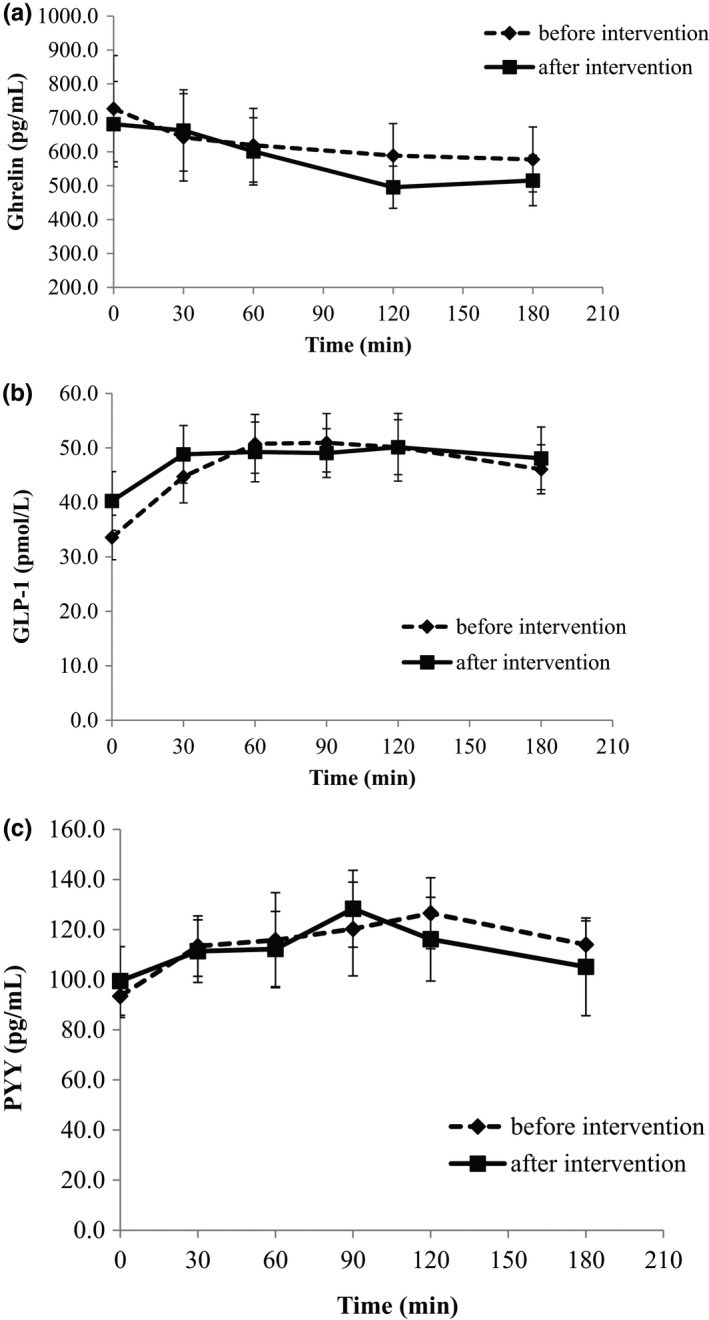
Absolute concentrations of plasma ghrelin (a), GLP‐1 (b), and PYY (c) during the mixed meal tolerance test in T2DM patients before and after the 12‐week dietary intervention. Values are presented as means ± SEM

Total ghrelin was reduced after consumption of the mixed meal, with a minimum value at 120 min postprandially both before and after the intervention. There was a tendency for a larger reduction of ghrelin levels after intervention; however, no significant difference between the two tests was observed (Figure 2a). Similarly, GLP‐1 and PYY responses did not show any change at the end of the study compared with the beginning (*p* > .05 for each time point and for AUC between the two mixed meal tolerance tests, Table 2; Figure 2b,c).

### 3.2 Subjective appetite ratings

**FIGURE 3:**
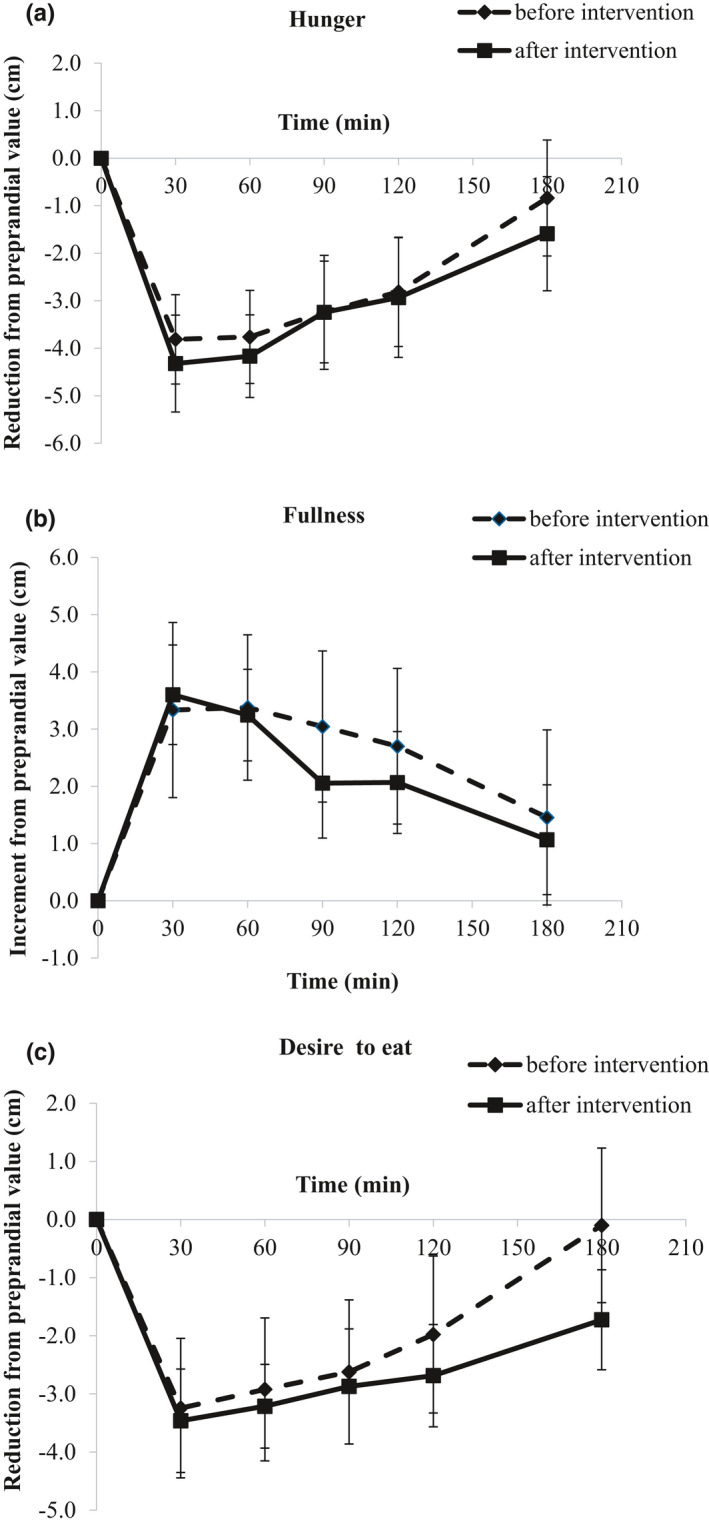
Subjective appetite ratings for hunger (a), fullness (b), and desire to eat (c) during the mixed meal tolerance test before and after the 12‐week dietary intervention. Values are presented as means ± SEM

Subjective appetite ratings are presented as differences from preprandial state over the 180 min after consumption of the meal, in Figure 3. iAUC values did not indicate any significant differences (Table 3).

**TABLE 3:** Incremental area under the curve (iAUC) for subjective appetite ratings (hunger, fullness, and desire to eat) before and after the dietary intervention with non‐fat, conventional yogurt

iAUC (cm × 180 min)	Baseline	End
Hunger	−507.43 ± 159.23	−573.65 ± 159.18
Fullness	436.83 ± 171.86	327.48 ± 102.00
Desire to eat	−483.52 ± 171.54	−431.54 ± 146.58

Note

Values are expressed as mean ± SEM.

## 4 DISCUSSION

The present study investigated the effects of a 12‐week, non‐energy‐restricted dietary intervention with non‐fat, conventional yogurt, on appetite hormone responses of overweight T2DM patients. Although there is a lot of discussion on the role of yogurt consumption in metabolic regulation, there is severe lack of evidence regarding the effect of yogurt on appetite especially in T2DM patients.

High intake of dairy products for 24 weeks (diet with 3 servings of dairy/day contributing with 1200 mg Ca/day) did not cause any reduction of body weight in overweight/obese subjects following a non‐energy‐restricted diet but resulted in decreased total body fat and increased lean body mass when compared to low dairy (<1 serving/day)/Ca (<500 mg/day) intake (Zemel et al., 2005) It has also been reported that dairy product consumption for a least period of 16 weeks has modest benefits on body weight and composition in overweigh/obese adults aged 18–50 years following energy‐restricted diets (Stonehouse et al., 2016).

Hunger‐reducing effects and beneficial changes in gastrointestinal hormones have been reported (Gilbert et al., 2011; Jones et al., 2013; Velhorst et al., 2008) in overweight/obese individuals consuming dairy while in weight‐reducing programs. However, these effects were not always accompanied by greater weight loss (Jones et al., 2013). Parameters such as the duration of the intervention, the type of dairy, and the study population cause variances in research results.

Especially for yogurt, high‐protein yogurt afternoon snacks have been shown to increase satiety compared to ones with lower protein content (Douglas et al., 2013; El Khoury et al., 2014) or to high‐fat snacks such as crackers and chocolate (Ortinau et al., 2014) contributing to lower subsequent energy intake. However, when plain yogurt with high protein content (23.1 g proteins/141 kcal) was administered to healthy males, no differences in subjective satiety ratings were noticed compared to strawberry yogurt with low protein content (18.3 g protein/167 kcal) (El Khoury et al., 2014). Additionally, no added benefit was observed in increasing lean mass by yogurt consumption and modest energy restriction, in overweight women followed a resistance‐training program for 16 weeks (Thomas et al., 2011).

In the present study, a non‐fat conventional yogurt was examined. The results demonstrated that intake of this type of yogurt for 12 weeks under non‐energy‐restricted diet did not affect appetite hormone responses, energy intake, or body weight. As it was mentioned before, high dairy intake by overweight/obese subjects (low dairy consumers) following a weight maintenance diet for 24 weeks did not change body weight but caused a significant reduction in total body fat and an increase in lean mass (Zemel et al., 2005). Two meta‐analyses have concluded that dairy consumption in situations without energy restriction neither leads to gain nor to loss of body weight (Abargouei et al., 2012; Chen et al., 2012). Beneficial effects on body composition were not observed in the present study, probably due to the smaller duration of the intervention. However, no significant association between total yogurt consumption and reversion of abdominal obesity status or lower waist circumference was found in elderly with high cardiovascular risk (Santiago et al., 2016).

Studies support the positive effects of whey protein and casein, the major fractions in dairy products on appetite hormones such as GLP‐1, PYY and cholecystokinin as well as ghrelin (Akhavan et al., 2010, 2014; Bowen et al., 2006). Even so, after a 12‐week energy‐restricted dietary intervention with high dairy and Ca in overweight/obese adults, a modest increase in plasma PYY concentrations during a mixed meal tolerance test was noticed but no other changes were observed regarding appetite hormone responses that is, GLP‐1, GIP, or ghrelin (Jones et al., 2013). In accordance with these findings, the present study demonstrated no differences in ghrelin, PYY, and GLP‐1 responses to a mixed meal tolerance test in overweight T2DM patients. These findings are also supported by subjective appetite ratings.

Although the number of participants is small and this is a limitation of the present study, the results are clear and support the findings of other relevant studies.

Considering the above‐mentioned results, it is concluded that conventional yogurt consumption for 12‐week duration without energy restriction does not affect appetite hormone responses and neither leads to gain nor to loss of body weight in overweight patients with T2DM. These findings are in accordance with those of studies conducted in non‐diabetic overweight/obese subjects with high dairy consumption for 12‐week interventions. More and well‐designed studies are needed in order to shed light on the underlying mechanisms regarding the effects of yogurt consumption on appetite regulation and implication of gastrointestinal hormones. Factors such as changing dietary habits by replacement of less healthy foods, the secretory effects of specific nutrients as well as the presence of lactic acid bacteria and their influence in shaping gut microbiota should be taken into consideration. It seems that the approach of yogurt fortification that is, with vitamin D or vitamins B (Heravifard et al., 2013; Jafari et al., 2016; Nikooyeh et al., 2014; Yanni et al., 2019) as well as probiotics (Qu et al., 2018; Tazakori et al., 2017) could be effective. Greater improvement in body composition and metabolic parameters was observed after consumption of yogurt fortified with whey protein, Ca, vitamin D, prebiotics, and probiotics compared with low‐fat plain yogurt under energy‐restricted diet (Mohammadi‐Sartang et al., 2018; Yanni et al., 2020).

## CONFLICTS OF INTEREST

There are no conflicts of interest to declare.

## AUTHOR CONTRIBUTIONS

**Amalia Yanni:** Conceptualization (equal); Data curation (equal); Writing‐original draft (lead); Writing‐review & editing (equal). **Panagiotis Konstantopoulos:** Investigation (equal). **Kleio Kartsioti:** Investigation (equal). **Panagiota Binou:** Investigation (equal). **Vaios Karathanos:** Conceptualization (equal). **Artemis Chatzigeorgiou:** Funding acquisition (equal). **Alexander Kokkinos:** Data curation (equal); Supervision (equal); Writing‐review & editing (equal).

## Data Availability

Data available on request from the authors.

## References

[fsn32606-bib-0001] Abargouei, A. S. , Janghorbani, M. , Salehi‐Marzijarani, M. , & Esmaillzadeh, A. (2012). Effect of dairy consumption on weight and body composition in adults: A systematic review and meta‐analysis of randomized controlled clinical trials. International Journal of Obesity, 36(12), 1485–1493. 22249225 10.1038/ijo.2011.269

[fsn32606-bib-0002] Akhavan, T. , Luhovy, B. L. , Brow, P. H. , Cho, C. E. , & Anderson, G. H. (2010). Effect of premeal consumption of whey protein and its hydrolysate on food intake and postmeal glycemia in insulin responses in young adults. The American Journal of Clinical Nutrition, 91, 966–975. 20164320 10.3945/ajcn.2009.28406

[fsn32606-bib-0003] Akhavan, T. , Luhovyy, B. L. , Panahi, S. , Kubant, R. , Brown, P. H. , & Anderson, G. H. (2014). Mechanism of action of pre‐meal consumption of whey protein and glycemic control in young adults. The Journal of Nutritional Biochemistry, 25, 36–43. 24314863 10.1016/j.jnutbio.2013.08.012

[fsn32606-bib-0004] Alvarez‐Bueno, C. , Cavero‐Redondo, I. , Martinez‐Vizcaino, V. , Sotos‐Prieto, M. , Ruiz, J. R. , & Gil, A. (2019). Effects of milk and dairy product consumption on type 2 diabetes: Overview of systematic reviews and meta‐analyses. Advances in Nutrition, 10(suppl_2), S154–S163. 10.1093/advances/nmy107 31089734 PMC6518137

[fsn32606-bib-0005] Bowen, J. , Noakes, M. , & Cliftron, P. M. (2006). Appetite regulatory hormone responses to various dietary proteins differ by body mass index status despite similar reductions in ad libitum energy intake. The Journal of Clinical Endocrinology & Metabolism, 91, 2913–2919. 16735482 10.1210/jc.2006-0609

[fsn32606-bib-0006] Chen, M. , Pan, A. , Malik, V. S. , & Hu, F. B. (2012). Effects of dairy intake on body weight and fat: A meta‐analysis of randomized controlled trials. The American Journal of Clinical Nutrition, 96, 735–747. 10.3945/ajcn.112.037119 22932282 PMC3441106

[fsn32606-bib-0007] Dougkas, A. , Reynolds, C. K. , Givens, I. D. , Elwood, P. C. , & Minihane, A. M. (2011). Associations between dairy consumption and body weight: A review of the evidence and underlying mechanisms. Nutrition Research Reviews, 24(1), 72–95. 21320381 10.1017/S095442241000034X

[fsn32606-bib-0008] Douglas, S. M. , Ortinau, L. , Hoertel, H. A. , & Leidy, H. J. (2013). Low, moderate, or high protein yogurt snacks on appetite control and subsequent eating in healthy women. Appetite, 60, 117–122. 10.1016/j.appet.2012.09.012 23022602

[fsn32606-bib-0009] El Khoury, D. , Brown, P. , Smith, G. , Berengut, S. , Panahi, S. , Kubant, R. , & Anderson, G. H. (2014). Increasing protein to carbohydrate ratio in yogurts consumed as a snack reduces post‐consumption glycemia independent of insulin. Clinical Nutrition, 33, 29–38. 23591152 10.1016/j.clnu.2013.03.010

[fsn32606-bib-0010] Gilbert, J. A. , Joanisse, D. R. , Chaput, J. P. , Miegueu, P. , Cianflone, K. , Alméras, N. , & Tremblay, A. (2011). Milk supplementation facilitates appetite control in obese women during weight loss: A randomized, single‐blind, placebo controlled trial. British Journal of Nutrition, 105, 133–143. 10.1017/S0007114510003119 21205360

[fsn32606-bib-0011] Guo, J. , Givens, D. I. , Astrup, A. , Bakker, S. J. L. , Goossens, G. H. , Kratz, M. , Marette, A. , Pijl, H. , & Soedamah‐Muthu, S. S. (2019). The impact of dairy products in the development of type 2 diabetes: Where does the evidence stand in 2019? Advances in Nutrition, 10(6), 1066–1075. 31124561 10.1093/advances/nmz050 PMC6855942

[fsn32606-bib-0012] Heravifard, S. , Neyestani, T. R. , Nikooyeh, B. , Alavi‐Majd, H. , Houshiarrad, A. , Kalayi, A. , Shariatzadeh, N. , Zahedirad, M. , Tayebinejad, N. , Salekzamani, S. , Khalaji, N. , & Gharavi, A. (2013). Regular consumption of both vitamin D‐ and calcium‐ and vitamin D‐fortified yogurt drink is equally accompanied by lowered blood lipoprotein (a) and elevated apoprotein A1 in subjects with type 2 diabetes: A randomized clinical trial. Journal of the American College of Nutrition, 32(1), 26–30. 23875809 10.1080/07315724.2013.767659

[fsn32606-bib-0013] Jafari, T. , Faghihimani, E. , Feizi, A. , Iraj, B. , Javanmard, S. H. , Esmaillzadeh, A. , Fallah, A. A. , & Askari, G. (2016). Effects of vitamin D‐fortified low fat yogurt on glycemic status, anthropometric indexes, inflammation, and bone turnover in diabetic postmenopausal women: A randomized controlled clinical trial. Clinical Nutrition, 35(1), 67–76. 25794439 10.1016/j.clnu.2015.02.014

[fsn32606-bib-0014] Jones, K. W. , Eller, L. K. , Parnell, J. A. , Doyle‐Baker, P. K. , Edwards, A. L. , & Reimer, R. A. (2013). Effect of dairy‐ and calcium‐rich diet on weight loss and appetite during energy restriction in overweight and obese adults: A randomized trial. European Journal of Clinical Nutrition, 67, 371–376. 23462943 10.1038/ejcn.2013.52 PMC3948984

[fsn32606-bib-0015] Mohammadi‐Sartang, M. , Bellissimo, N. , Totosy de Zepetnek, J. O. , Brett, N. R. , Mazloomi, S. M. , Fararouie, M. , Bedeltavana, A. , Famouri, M. , & Mazloom, Z. (2018). The effect of daily fortified yogurt consumption on weight loss in adults with metabolic syndrome: A 10‐week randomized controlled trial. Nutrition, Metabolism and Cardiovascular Diseases, 28(6), 565–574. 10.1016/j.numecd.2018.03.001 29724529

[fsn32606-bib-0016] Nikooyeh, B. , Neyestani, T. R. , Tayebinejad, N. , Alavi‐Majd, H. , Shariatzadeh, N. , Kalayi, A. , Zahedirad, M. , Heravifard, S. , & Salekzamani, S. (2014). Daily intake of vitamin D‐ or calcium‐vitamin D‐fortified Persian yogurt drink (doogh) attenuates diabetes‐induced oxidative stress: Evidence for antioxidative properties of vitamin D. Journal of Human Nutrition and Dietetics, 27(Suppl 2), 276–283. 23829785 10.1111/jhn.12142

[fsn32606-bib-0017] Ortinau, L. C. , Hoertel, H. A. , Douglas, S. M. , & Leidy, H. J. (2014). Effects of high protein vs. high fat snacks on appetite control, satiety, and eating initiation in healthy women. Nutrition Journal, 13, 97–101. 10.1186/1475-2891-13-97 25266206 PMC4190484

[fsn32606-bib-0018] Panahi, S. , Luhovyy, B. L. , Liu, T. T. , Akhavan, T. , El Khoury, D. , Goff, H. D. , & Harvey Anderson, G. (2013). Energy and macronutrient content of familiar beverages interact with pre‐meal intervals to determine later food intake, appetite and glycemic response in young adults. Appetite, 60, 154–161. 10.1016/j.appet.2012.09.018 23022554

[fsn32606-bib-0019] Panahi, S. , & Tremblay, A. (2016). The potential role of yoghurt in weight management and prevention of type 2 diabetes. Journal of the American College of Nutrition, 0, 1–15. 10.1080/07315724.2015.1102103 27332081

[fsn32606-bib-0020] Qu, L. , Ren, J. , Huang, L. , Pang, B. , Liu, X. , Liu, X. , Li, B. , & Shan, Y. (2018). Antidiabetic effects of *Lactobacillus casei* fermented yogurt through reshaping gut microbiota structure in type 2 diabetic rats. Journal of Agricultural and Food Chemistry, 66(48), 12696–12705. 30398060 10.1021/acs.jafc.8b04874

[fsn32606-bib-0021] Santiago, S. , Sayón‐Orea, C. , Babio, N. , Ruiz‐Canela, M. , Martí, A. , Corella, D. , Estruch, R. , Fitó, M. , Aros, F. , Ros, E. , Gómez‐García, E. , Fiol, M. , Lapetra, J. , Serra‐Majem, L. , Becerra‐Tomás, N. , Salas‐Salvadó, J. , Pinto, X. , Schröder, H. , & Martínez, J. A. (2016). Yogurt consumption and abdominal obesity reversion in the PREDIMED study. Nutrition, Metabolism and Cardiovascular Diseases, 26(6), 468–475. 10.1016/j.numecd.2015.11.012 26988650

[fsn32606-bib-0022] Stonehouse, W. , Wycherley, T. , Luscombe‐Marsh, N. , Taylor, P. , Brinkworth, G. , & Riley, M. (2016). Dairy intake enhances body weight and composition changes during energy restriction in 18–50‐year‐old adults‐a meta‐analysis of randomized controlled trials. Nutrients, 8(7), 394. 10.3390/nu8070394 PMC4963870 27376321

[fsn32606-bib-0023] Tazakori, Z. , Zare, M. , & Jafarabadi, M. A. (2017). Probiotic yogurt effect on macronutrients ingredients, blood glucose and lipid profile in type 2 diabetes. Journal of Pakistan Medical Association, 67(7), 1123. 28770903

[fsn32606-bib-0024] Thomas, D. T. , Wideman, L. , & Lovelady, C. A. (2011). Effects of dairy supplement and resistance training on lean mass and insulin‐like growth factor in women. International Journal of Sport Nutrition and Exercise Metabolism, 21, 181–188. 21719898 10.1123/ijsnem.21.3.181

[fsn32606-bib-0025] Tremblay, A. , Doyon, C. , & Sanchez, M. (2015). Impact of yoghurt on appetite control, energy balance, and body composition. Nutrition Reviews, 73, 23–27. 26175486 10.1093/nutrit/nuv015

[fsn32606-bib-0026] Velhorst, M. , Smeets, A. , Soenen, S. , Hochstenbach‐Waelen, A. , Hursel, R. , Diepvens, K. , Lejeune, M. , Luscombe‐Marsh, N. , & Westerterp‐Plantenga, M. (2008). Protein‐induced satiety: Effects and mechanisms of different proteins. Physiology & Behavior, 94, 300–307. 10.1016/j.physbeh.2008.01.003 18282589

[fsn32606-bib-0027] Yanni, A. E. , Kartsioti, K. , & Karathanos, V. T. (2020). The role of yoghurt consumption in the management of type II diabetes. Food & Function, 11, 10306–10316. 10.1039/D0FO02297G 33211046

[fsn32606-bib-0028] Yanni, A. E. , Kokkinos, A. , Psychogiou, G. , Binou, P. , Kartsioti, K. , Chatzigeorgiou, A. , Konstantopoulos, P. , Perrea, D. , Tentolouris, N. , & Karathanos, V. T. (2019). Daily consumption of fruit‐flavored yoghurt enriched with vitamins B contributes to lower energy intake and body weight reduction, in type 2 diabetic patients: A randomized clinical trial. Food & Function, 10(11), 7435–7443. 31663569 10.1039/c9fo01796h

[fsn32606-bib-0029] Yanni, A. E. , Stamataki, N. S. , Stoupaki, M. , Konstantopoulos, P. , Pateras, I. , Tentolouris, N. , Perrea, D. , & Karathanos, V. T. (2017). Cr‐enriched yeast: Beyond fibers for the management of postprandial glycemic response to bread. European Journal of Nutrition, 56(4), 1445–1453. 26913854 10.1007/s00394-016-1190-4

[fsn32606-bib-0030] Zemel, M. B. , Richards, J. , Milstead, A. , & Campbell, P. (2005). Effects of calcium and dairy on body composition and weight loss in African‐American adults. Obesity Research, 13(7), 1218–1225. 16076991 10.1038/oby.2005.144

